# Conservative management of abnormally invasive placenta complicated by local hyperfibrinolysis and beginning disseminated intravascular coagulation

**DOI:** 10.1007/s00404-020-05721-0

**Published:** 2020-08-18

**Authors:** C. Biele, L. Kaufner, A. Schwickert, A. Nonnenmacher, K. von Weizsäcker, M. Z. Muallem, W. Henrich, T. Braun

**Affiliations:** 1 Department of Obstetrics, Charité - Universitätsmedizin Berlin, Corporate Member of Freie Universität Berlin, Humboldt-Universität zu Berlin, and Berlin Institute of Health, Augustenburger Platz 1, 13353 Berlin, Germany; 2 Department of Anesthesiology and Operative Intensive Care Medicine, Charité - Universitätsmedizin Berlin, Corporate Member of Freie Universität Berlin, Humboldt-Universität zu Berlin, and Berlin Institute of Health, Augustenburger Platz 1, 13353 Berlin, Germany; 3 Department of Gynecology With Center of Oncological Surgery, Charité - Universitätsmedizin Berlin, Corporate Member of Freie Universität Berlin, Humboldt-Universität zu Berlin, and Berlin Institute of Health, Augustenburger Platz 1, 13353 Berlin, Germany; 4 Department of ‘Experimental Obstetrics’ and Study Group ‘Perinatal Programming’, Charité - Universitätsmedizin Berlin, Corporate Member of Freie Universität Berlin, Humboldt-Universität zu Berlin, and Berlin Institute of Health, Augustenburger Platz 1, 13353 Berlin, Germany

**Keywords:** Placenta percreta, Abnormal invasive placenta, Conservative management, Placenta in situ, Disseminated intravascular coagulation, Fibrinogen levels

## Abstract

**Introduction:**

Abnormally invasive placenta (AIP) is often associated with high maternal morbidity. In surgical treatment, caesarean hysterectomy or partial uterine resection may lead to high perioperative maternal blood loss. A conservative treatment by leaving the placenta in utero after caesarean delivery of the baby is an option to preserve fertility and to reduce peripartum hysterectomy-related morbidity. Nevertheless, due to increased placental coagulation activity as well as consumption of clotting factors, a disseminated intravascular coagulation (DIC)-like state with secondary late postpartum bleeding can occur.

**Purpose:**

Systematic review after the presentation of a case of conservative management of placenta percreta with secondary partial uterine wall resection due to vaginal bleeding, complicated by local hyperfibrinolysis and consecutive systemic decrease in fibrinogen levels.

**Methods:**

Systematic PubMed database search was done until August 2019 without any restriction of publication date or journal

**Results:**

Among 58 publications, a total of 11 reported on DIC-like symptoms in the conservative management of AIP, in the median on day 59 postpartum. In most cases, emergency hysterectomy was performed, which led to an almost immediate normalization of coagulation status but was accompanied with high maternal blood loss. In two cases, fertility-preserving conservative management could be continued after successful medical therapy.

**Conclusion:**

Based on these results, we suggest routinely monitoring of the coagulation parameters next to signs of infection in the postpartum check-ups during conservative management of AIP. Postpartum tranexamic acid oral dosage should be discussed when fibrinogen levels are decreasing and D-Dimers are increasing.

**Electronic supplementary material:**

The online version of this article (10.1007/s00404-020-05721-0) contains supplementary material, which is available to authorized users.

## Introduction

The condition of abnormal invasive placenta (AIP) and especially placenta percreta is a rare, but due to high maternal morbidity, very important and present problem of obstetric care. With increasing rates of caesarean section (CS), there is also an increasing incidence of placenta percreta [1].

Between 2000 and 2015, the global CS rate has almost doubled (12.1% of all births in 2000 vs. 21.1% in 2015) [2]. A large American study showed the increasing incidence of intrapartum hysterectomy (HE, in most of the cases attributed to AIP) with every prior CS. In a pregnancy, after the first CS, 0.64% of the patients had HE, after the third CS, HE was performed in 2.4% due to AIP. In cases of placenta praevia, the risk for AIP was even higher (3% after one CS, 11% after two CSs, 40% after three, 60% after four, and increasing with each CS) [2].

In a case of AIP, caesarean hysterectomy is considered the gold standard procedure, even though it is associated with high rates of severe maternal morbidity (40–50%) and mortality (7%) [3]. To reduce maternal morbidity (especially massive hemorrhage), and to preserve fertility [4], conservative management with leaving the placenta in situ has become more common again over the last years [3, 5]. After caesarean delivery without removing the placenta, the blood flow in the placenta decreases significantly. This leads over time to necrosis and spontaneous placental detachment from the uterus and even other invaded organs [3]. Therefore, this method is considered an alternative, especially for severe cases of AIP [5]. Nevertheless, there are also severe possible complications that can occur in this therapeutic concept, such as infection, sepsis and massive vaginal hemorrhage, even accompanied by DIC [6]. Here, we present a case of conservative management of placenta percreta complicated by symptomatic local placental hyperfibrinolysis and fibrinogen consumption with secondary bleeding on day 54 postpartum and a high risk of DIC and provide a systematic review of the current literature focused on hemostatic changes and DIC in conservative management of AIP.

## Case presentation

**Fig. 1:**
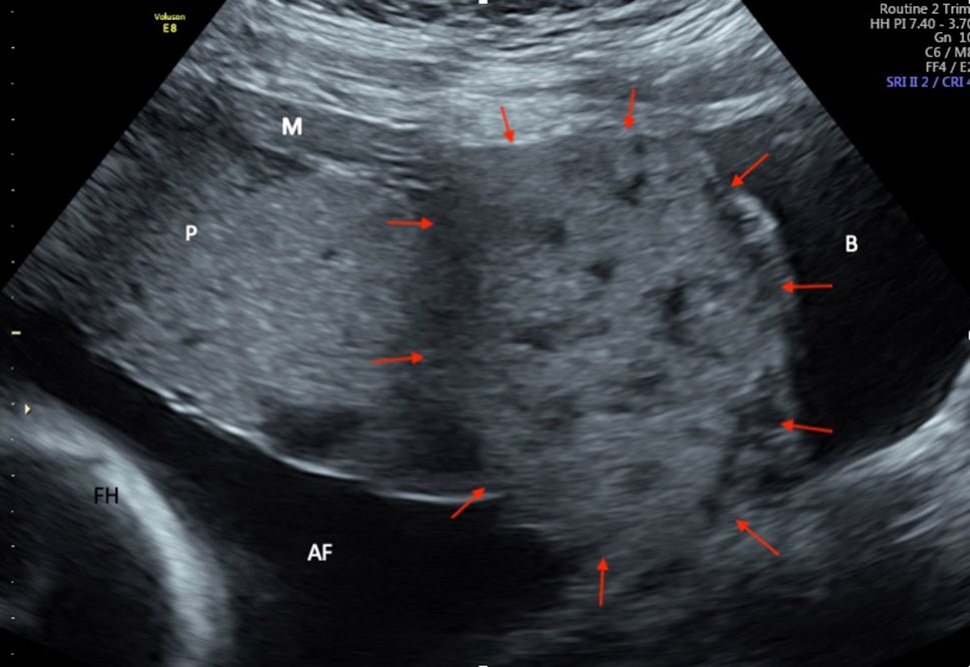
Abdominal sonography in 22 + 6 weeks of gestation, sagittal view, b-mode. Suspected percrete area of the placenta (marked with arrows) with typical bulging into the bladder (B) and thinning of the myometrium (M). Fetal head (FH), placenta (P), amniotic fluid (AF)

**Fig. 2:**
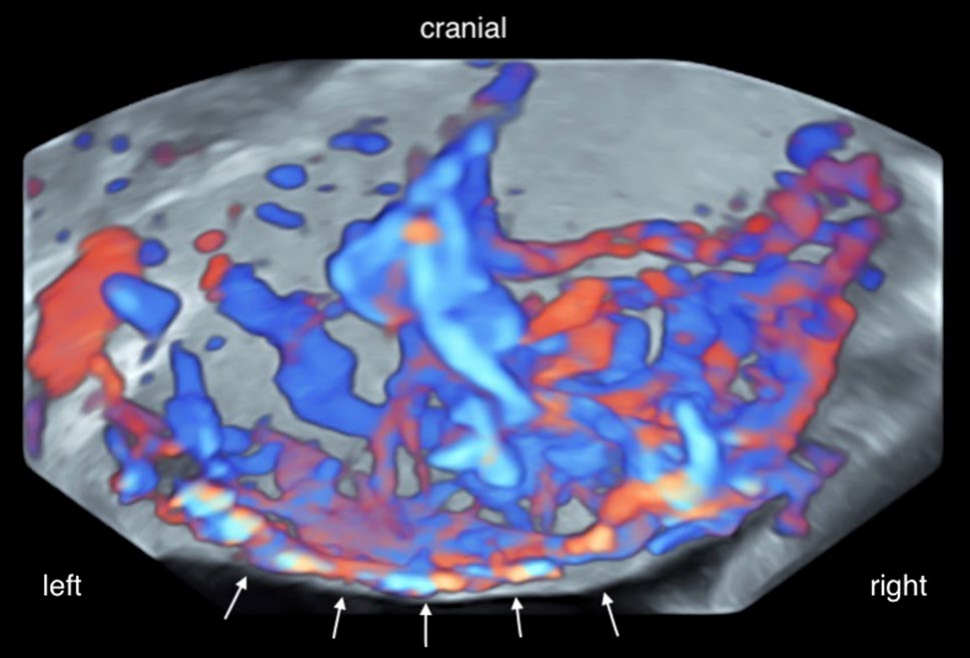
Transvaginal sonography in 22 + 6 weeks of gestation, colour mode, glassbody mode. Pattern of vascularisation at the interface between placenta and bladder wall with bridging vessels. Bladder wall is marked with arrows

A healthy 37 year old IV gravida II para came to our hospital in 22 + 6 weeks of pregnancy for a second opinion with suspected placenta percreta. She has had two prior caesarean sections (the first one due to breech presentation, the second one planned repeated) and one early abortion followed by curettage. In ultrasound, the placenta praevia totalis of the anterior uterine wall showed distinct signs of placenta percreta with suspected bladder invasion. No myometrium between placenta and bladder and very irregular patterns of perfusion were observed, such as large lacunas and bridging vessels in the interface (Figs. 1 and 2). The MRI confirmed the suspected placenta percreta with no valid signs of invading other organs. She was counseled in detail about the serious risks of this diagnosis. Termination of the pregnancy was not an option for our patient. She had the urgent wish to preserve fertility due to unfinished family planning with a new partner. Segmental excision (if possible) or leaving the placenta in situ was planned. For the case of emergency, we also had her consent for hysterectomy, though. At 34 + 5 weeks of gestation, she presented with preterm contractions, secondary caesarean section through a fundal uterine incision after amniotic drainage and exteriorization of the gravid uterus [7] was performed in combined neuraxial anesthesia and general anesthesia after birth without complications. Due to high risk of intraoperative bleeding, an arterial line and two 16 G peripheral vein cannulas were placed. Additionally, red blood cell concentrates as well as fresh frozen plasma were provided in theatre and cell savage, and norepinephrine was prepared in case of massive bleeding. Prophylactic tranexamic acid was applied continuously in a dosage of 15 mg/kg/bw during surgery without complications. A healthy boy of 2800 g, Apgar 8/8/8, NapH 7.31 was delivered, estimated blood loss was 500 ml. Due to extensive size of at least 10 × 8 cm of the percrete area, the placenta was left in situ. No transfusions of blood or hemostatic products were necessary and no further postpartum hemorrhage occurred.

The postoperative course remained without complications. She received an antibiotic prophylaxis with metronidazole and cefuroxime, regular blood tests for inflammation parameters and cervical swabs for bacteria were taken and showed no signs of infection, no vaginal hemorrhage occurred. She was discharged into ambulant care on day seven; weekly check-ups for infection and the antibiotic prophylaxis (400 mg of metronidazole two times a day, 500 mg of cefuroxime three times a day, oral application) were continued. Matching the antibiogram of bacteria from cervical swabs, the antibiotic therapy was changed to amoxicillin in the further course.

On day 54, she presented with vaginal bleeding. Blood tests still showed no signs of infection, in ultrasound, the placenta was in situ with reduced perfusion.

**Fig. 3:**
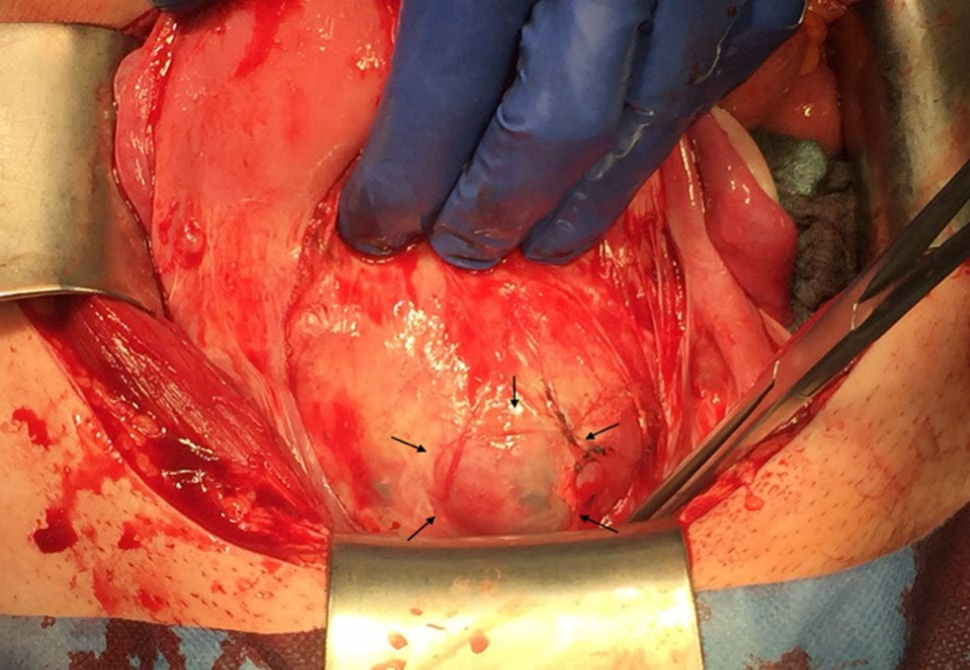
Intraoperative image, day 55 postpartum. Anterior uterine wall before the partial resection with the placenta still in situ and the percrete area visible from the outside (marked with arrows) after surgical preparation and separation of the adhesive bladder wall. The bladder wall itself was not invaded by placenta tissue, as suspected in ultrasound

**Fig. 4:**
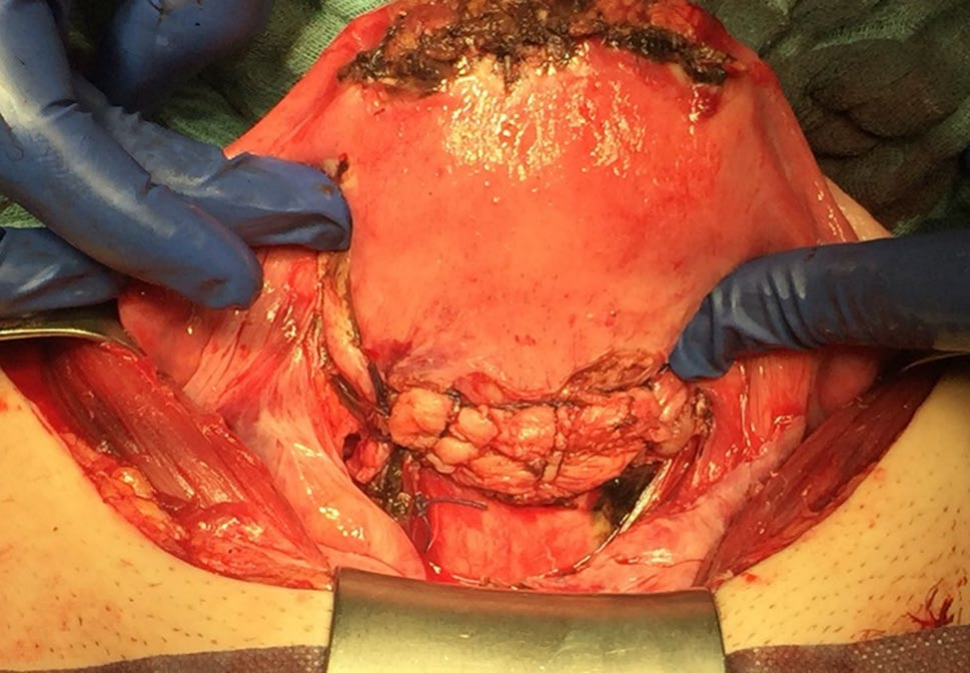
Intraoperative image, day 55 postpartum. Anterior uterine wall after partial uterine resection and reconstruction. Cranial visible the fundal incision from the cesarean section

The different treatment options were discussed; embolism of the uterine arteries, hysterectomy vs. segmental uterine resection (if possible) or expectant management. On ultrasound examination, the uterus and also the percrete area presented significantly smaller, a surgical approach with uterine preservation (if possible) was planned. The preoperative blood work-up showed a reduced Quick value of 41%, increased d-dimere levels > 20 mg/l (normal range < 0.5 mg/l) and reduced fibrinogen levels of 0.59 g/l (normal range 1.6–4 g/l), so that 1 g of tranexamic acid was administered preoperatively. The hemoglobin level was 13.6 g/dl. Segmental uterine resection was performed and the uterus could be reconstructed. The bladder was not invaded as suspected in ultrasound, but surgical preparation and separation of the bladder wall from placental tissue resulted in a small bladder wall lesion which had to repaired (Figs. 3 and 4).

**Fig. 5:**
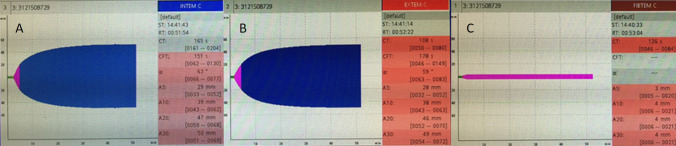
**a**–**c** Rotational Thromboelastometric analysis in citrated blood samples (ROTEM®, TEM International, Munich, Germany) showing the clot formation (mm) over time (s) at the beginning of surgery. The INTEM® (phospholipids and ellagic acid to initiate intrinsic clotting; (**a**) and EXTEM® (tissue factor to initiate extrinsic clotting; (**b**) analysis with prolonged clot formation times (CFT, seconds) and reduced clotting amplitudes after a clotting time (CT, seconds) of 5, 10, 20, 30 min (A5-A30; mm) and clot formation angle (α, degrees) reflect the concomitant consumption of clotting factors for the intrinsic and extrinsic pathway of coagulation. The reduced amplitudes of the fibrinogen related clot formation at different time points (A5-A30) as well as the prolonged CT in the FIBTEM® Test (Inhibition of platelet activity using cytochalasin to reflect clot tracing dependent on fibrinogen; (**c**) show the reduced functional fibrinogen fraction in clot formation

Right from the beginning of the surgery, a diffuse bleeding tendency was observed. The thromboelastometric analysis (ROTEM®, TEM International, Munich, Germany) at the beginning of surgery showed a prolonged clotting time (CT: 126 s (46–83 s)) as well as reduced amplitudes of the fibrinogen related clot formation at different time points [A5: 3 (5–30) mm; A10: 4 (6–21) mm; A20: 4 (6–21) mm; A30: 4 (6–21) mm] in the FIBTEM® Test (Fig. 5). These results are in line with the reduced fibrinogen levels of the preoperative blood work-up. Furthermore, the INTEM® and EXTEM® analysis with prolonged clot formation times [CFT INTEM®: 151 (62–130) s; CFT EXTEM®: 178 (46–149) s.], the reduced clotting amplitudes at all time points (A5–A30; Fig. 5) as well as the reduced Quick value of 41% in the preoperative blood work-up indicate an impaired coagulation due to fibrinogen consumption and a beginning DIC with a concomitant consumption of clotting factors (Fig. 5) [8]. Hyperfibrinolysis could not be detected; TXA had been administered before, though (Fig. 5). Nevertheless, an increased d-dimere level > 20 mg/l in the preoperative blood work-up suggests a sign of initially increased local hyperfibrinolysis before TXA was given. In consideration of the thromboelastometric analysis and the results of the blood work-up accompanied with ongoing intraoperative bleeding, two red blood cell concentrates (RBC) at a further decreasing hemoglobin level of 6.8 g/dl, three fresh frozen plasma units (FFP), prothrombin complex concentrate (PSSB, 1800 IE), 4 g of fibrinogen and tranexamic acid [15 mg/kg body weight (bw)] were administered until the bleeding could be controlled. A continuous application of norepinephrine in a dosage up to 0.2 μg/kg bw/min was temporally necessary for hemodynamic stabilization. Estimated blood loss was 3500 ml.

After surgery, the patient was admitted to the intensive care unit for 24 h without any signs of recurrent bleeding or cardiorespiratory limitations. Coagulation parameters had already normalized 8 h after the operation (Quick value 87%, fibrinogen 2.41 g/l) and the hemoglobin levels remained stable (around 8 g/dl). On day seven, the cystogram showed no persistent bladder lesion, the catheter could be removed. The patient was discharged on the seventh postoperative day (day 62 postpartum) in good health condition. Due to segmental resection of the uterine wall, we did not recommend a pregnancy for the next two years.

## Review of the literature

A PubMed database search in English or German was performed without any restriction of publication date or journal on the complication of DIC in the conservative management of placenta percreta. Keywords: conservative management, abnormal invasive placenta, placenta percreta, leaving the placenta in situ, DIC, coagulopathy. The last search was updated in August 2019. All references of the selected articles were hand-searched for relevant studies not captured by electronic searches. Cases reported more than once by the same team in different publications were counted only once. Individually reported cases were not recounted in the larger case series. After removing duplicates, 58 papers were identified and finally eight studies reporting about 11 cases with DIC- or DIC-like symptoms in conservative management of AIP were included (flow diagram supplement).

We found no prospective randomized or observational trial as well no evidence-based recommendations in the literature. DIC is a multifactorial clinicopathological syndrome with several possible triggers. It is mainly characterized by systemic activation of coagulation pathways, which lead to uncontrolled generation of fibrin clots and, due to consumption of coagulation factors, may cause severe bleeding [9]. Therefore, most of the cases switch from a conservative into surgical management postpartum. Only in two cases, conservative management could be continued [10, 11], whereas in six cases, secondary hysterectomy due two massive bleeding was performed [6, 12–15]. In three cases, no further specified secondary surgery was performed or listed [16] (Table 1).

**Table 1:** Overview of all 11 AIP cases with conservative approach and documented DIC

Author/cases	Diagnosis of DIC (day)	Symptoms	Treatment of DIC	Success of treatment	Preservation of the uterus
Judy et al. [12]/1	49	Hematoma Easy bruising Gingival bleeding Brown discharge	FFP (2) Cryoprecipitate	No	No (HE)
Bourellier et al. [13]/2	58	Spontaneous hematoma	RBC (2) FFP (2) Fibrinogen (2 g) Embolization of uterine arteries	No	No (emergency HE)
	60	Hematoma Hemarthrosis of the knee	Embolization of uterine arteries	No	No (emergency HE)
Desbriere et al. [14]/1	72	Spontaneous hematoma Brown discharge	RBC (n.s.) Embolization of uterine arteries	No	No (HE)
Wong et al. [15]/1	58	Hematoma of axilla with compression of radial nerve, (required surgery)	FFP (12) RBC (11) Octaplas (20) Cryoprecipitate (20)	No	No (HE)
Pather et al. [6]/1	120	Vaginal bleeding Fever	Embolization of uterine arteries Manual removal of the placenta	No	No (HE)
Matzusaki et al. [10]/1	46	No symptoms	First try: Unfractionated heparin FFP	No	
			Second try: APPT-adjusted intravenous heparin	Yes	Yes. Complete resorption of the placenta, day 121
Schröder et al. [11]/1	71	Vaginal bleeding Gingival bleeding Easy bruising	Tranexamic acid Enoxaparin	Yes	Yes Removal of placenta by curettage, day 119
H.-W. Su et al. [16]/3	36, 60, 63	Hematuria Diffuse skin bruise	n.s	n.s	n.s (“second surgical procedure”)

*APPT* activated partil thromboplastin time, *n.s.* not specified, *HE*, hysterectomy, *FFP* fresh frozen plasma, *RBC* red blood cell transfusion

Judy et al*.* presented a case of a patient with easy bruising, gingival bleeding and brown discharge seven weeks (day 49) postpartum [12]. Laboratory tests showed specific signs of DIC: low fibrinogen levels (46 mg/dl with a normal range of 236–389 mg/dl), high d-dimer (20.000 ng/ml, cut off 500 ng/dl) and prolonged partial thromboplastin and prothrombin time. The coagulation parameters had last been checked on day 6, when they were normal. The patient received two FFP´s and two cryoprecipitates, which led to a slight improvement of the coagulation parameters. On the next day, abdominal hysterectomy was performed and she received several blood products (5 FFP, 7 RBC, 3 platelets, 6 cryoprecipitate). On the next day, coagulation parameters had normalized.

Two cases are reported by Bourrellier et al., both patients presented with hematoma, one also with hemarthrosis of the knee, on day 58 and 60 [13]. In both patients, DIC was diagnosed. The first patient received 2 RBC`s, 2 FFP`s and 2 g of fibrinogen, which only led to a slight improvement, embolization of the uterine arteries was performed. The second patient received embolization right away. Both presented with severe vaginal hemorrhage within hours after embolization (6 and 48 h), emergency hysterectomy had to be performed in both cases.

In another case, Desbriere et al. presented a patient who developed spontaneous cutaneous bruises and brown vaginal discharge on day 72, blood tests showed a DIC (fibrinogen 0,6 with normal range 1,6–4 g/l, low platelets, prolonged APPT to 55 s) [14]. Due to progression of the DIC despite transfusion, hysterectomy was performed after embolization of the uterine and the hypogastric arteries. Estimated blood loss was about 5000 ml, several blood products were needed.

Wong et al*.* and Pather et al. each presented cases with symptoms of DIC that resolved after hysterectomy [6, 15]. Su et al. reported about three patients with DIC in the postpartum period who received non specified surgery [16]. In all cases, coagulation parameters normalized after removal of the placenta.

In two cases, fertility-preserving conservative management could be continued despite the occurrence of DIC. In the first case, Matsuzakis patient had a check-up every 14 days including coagulation parameter [10]. On day 42, her fibrinogen level had decreased to 114 mg/dl (normal range on this scale 150–350 mg/dl) and then to 62 mg/dl on day 46. Further tests confirmed DIC; the patient showed no symptoms. Secondary hysterectomy was planned, but the patient insisted on continuing conservatively. Unfractionated heparin and transfusion of FFP`s did not improve her fibrinogen levels. On day 48, anticoagulant therapy with intravenous unfractionated heparin was started and continued to day 69, the level of fibrinogen successfully improved. After that she developed fever but without signs of infections and only treated symptomatically. On day 121, the uterus was normal-sized with no residual placenta.

The second successful case was presented by Schröder et al. [11]. The patient was readmitted to the hospital on day 71 with vaginal and gingival bleeding and easy bruising, blood tests showed severe hyperfibrinolysis (64 mg/dl, normal range 150–450) and other sings of DIC. Therapy with tranexamic acid (1 g every 8 h) was started, and over the next days, the bleeding stopped and fibrinogen levels increased. For thromboembolic prophylaxis, she received enoxaparin (20 g per day). She remained symptom free until a slight vaginal bleeding occurred in day 119 but with normal coagulation parameters. Sonography showed partial separation of the placenta, uncomplicated curettage was performed.

## Discussion

DIC seems to be a rare but very serious complication in conservative management of placenta percreta. Nevertheless, the coagulation state in AIP before birth as well as hemostatic changes during a conservative therapy approach, are unknown. Systemic changes of coagulation in normal pregnancy and the endothelium like functions of the placenta-trophoblast in the third stage of labour leads to an increased coagulation and decreased fibrinolytic activity before birth [17, 18]. In AIP, the invasive growth of the placenta due to massive trophoblast invasion and enhanced trophoblast activity raises the suspicion, that local as well as systemic coagulation activity is more increased in women with placenta percreta. Therefore, a “DIC-like hemostatic state” is supposable. Depending on the degree of placental invasion and due to the massive syncytiotrophoblast invasion [19] with increased activity at the maternal- fetal interface and due to potential maternal endothelium dysfunction in the placenta or the surrounded placenta infiltrated organs the coagulation activity [19, 20] might be increased with first signs of consumption, but without clinical signs of DIC. Most of the authors reported a removal of the placenta by hysterectomy. On the one hand, immediate hysterectomy leads to an almost immediate normalization of all coagulation parameters postoperatively; on the other hand, high blood loss and massive transfusion occurred. In case of massive bleeding, coagulation may change rapidly into DIC and a targeted hemostatic therapy is required. One advantage for a conservative approach of AIP management might be to minimize the initial blood loss and the risk of immediate coagulation change into a DIC-like hemostatic state by leaving the placenta untouched. Furthermore, the still increased trophoblast induced enhanced local coagulation activity might lead to a massive consumption with a fibrinogen decrease, d-dimere increase as a marker of local fibrinolysis and with an increased risk of secondary postpartum hemorrhage [10–12, 21]. Therefore, beside the recommended FIGO guidelines check-up visits with clinical examination, pelvic ultrasound, blood tests for infection and vaginal swabs for bacteria [3] (weekly for a two month period, followed by monthly check-up visits until complete resorption), coagulation parameters, such as fibrinogen and d-dimer levels, should be closely monitored in the blood work-up up to 3 months postpartum. We therefore suggest tranexamic acid orally postpartum when fibrinogen levels are decreasing and D-Dimers are increasing. Based on the few cases published in the literature, the critical window for more intensive testing of the coagulation status is around 59 (median) days after delivery (95% CI 49–76 days), the estimated incidence of DIC is reported with 11% [6]. So far, there is no evidence-based concept for the follow-up after leaving the placenta in situ due to a lack of randomized controlled trials [3].

## Conclusion

Local hyperfibrinolysis and beginning disseminated intravascular coagulation are possible and serious complications in conservative management of AIP. Increased placental coagulation activity may lead to massive consumption with a fibrinogen decrease and d-dimere increase as a marker of local fibrinolysis and with a high risk of secondary postpartum hemorrhage. Therefore, regular blood tests of the coagulation parameters, such as fibrinogen and d-dimere, should be included into the check-up visits of the postpartum period for early diagnosis. The risk for coagulation changes seems to be the highest around day 59, check-ups should take place more frequently in this period. When diagnosed, oral dosage of tranexamic acid seems to be a possible therapy option.

## Electronic supplementary material

Below is the link to the electronic supplementary material.

Supplementary file1 (DOCX 68 kb)
