# Supplementary material for: Conservative management of abnormally invasive placenta complicated by local hyperfibrinolysis and beginning disseminated intravascular coagulation
Source: Arch Gynecol Obstet. 2020 Aug 18;303(1):61–8. doi: 10.1007/s00404-020-05721-0 (PMC7854425; doi:10.1007/s00404-020-05721-0)
Supplement: Supplementary file 1 — Supplementary file1 (DOCX 68 kb) [file 404_2020_5721_MOESM1_ESM.docx]

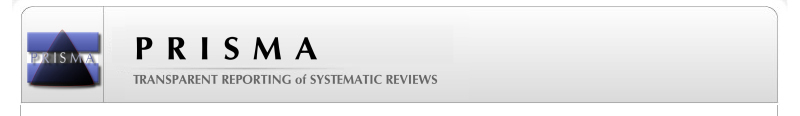
**PRISMA 2009 Flow Diagram**

Records excluded

(Cases reporting about DIC or conservative management of AIP, not in combination)

(n = 17)

Full-text articles excluded

(No further mention of diagnosis, symptoms or treatment of DIC)
(n =7)

Studies included in quantitative synthesis (meta-analysis)
(n = 8)

Studies included in qualitative synthesis
(n = 8)

Full-text articles assessed for eligibility
(n = 15)

Records screened
(n = 32)

Records after duplicates removed
(n = 58)

Additional records identified through other sources
(n = 0)

## Identification

## Eligibility

## Included

## Screening

Records identified through database searching
(n = 141)
